# Serotonin levels in aqueous humor of patients with primary open-angle glaucoma

**Published:** 2008-11-28

**Authors:** V. Zanon-Moreno, P. Melo, M.M. Mendes-Pinto, C.J. Alves, J.J. Garcia-Medina, I. Vinuesa-Silva, M.A. Moreno-Nadal, M.D. Pinazo-Duran

**Affiliations:** 1 Ophthalmologic Research Unit “Santiago Grisolia,” Dr. Peset University Hospital, Valencia, Spain; 2 IBMC-Instituto de Biologia Molecular e Celular, Universidade do Porto, Porto, Portugal; 3 Department of Ophthalmology, Punta de Europa Hospital, Algeciras, Cadiz, Spain; 4 Department of Ophthalmology, Dr. Peset University Hospital, Valencia, Spain

## Abstract

**Purpose:**

Glaucoma is an optic neuropathy characterized by a high intraocular pressure (IOP), alterations in optic nerve head, and loss of visual field that could lead to bilateral blindness. Serotonin (5-HT) is a biogenic monoamine that is synthesized from hydroxylation of tryptophan and acts by three ways, dissemination, metabolism, and reuptake in synaptic cleft through specific systems of the membrane. The purpose of this study is to evaluate the 5-HT and 5-HIAA (5-hydroxiindolacetic acid) levels in the aqueous humor of patients with primary open-angle glaucoma (POAG).

**Methods:**

We performed a case-control study, and the patients recruited were classified into two groups, 1) 30 patients with POAG (GG) and 2) 30 patients with cataracts (CG), who acted as the controls. Aqueous humor samples of each patient were obtained by paracentesis at the beginning of the surgical procedures. 5-HT and 5-HIAA levels were determined by high performance liquid chromatography (HPLC) with electrochemical detection.

**Results:**

There were no statistical differences between age (71.3±7.2 years in GG, 73.5±9.0 years in CG; p=0.2581) or gender (sex ratio 0.765 in GG and 0.667 in CG). 5-HT levels were lower in GG, but this difference was not significant (p=0.820). We observed a statistically significant higher level of 5-HIAA in GG (p=0.001). The 5-HT turnover (5-HIAA/5-HT) were higher in GG than in CG (p<0.05), but the difference was not significant (p=0.598).

**Conclusions:**

The level of 5-HT was lower in GG patients, and the level of 5-HIAA was higher in GG patients than in CG patients.

## Introduction

Glaucoma is one of the main causes of blindness, only behind cataracts. Its prevalence in the world is 2.4%, and there are approximately 105 million people with glaucoma worldwide [1,2]. This disease is characterized by a progressive loss of ganglion fibers, which involve the occurrence of the peripheral visual field loss [3].

There are many risk factors for glaucoma, and intraocular pressure (IOP) is the most important. That is why the treatment for this optic neuropathy always was led to lowering IOP [4]. Researchers have also been studying the mechanisms of cell death in glaucoma, and presently it is known that the treatment against ocular hypertension (OHT) alone is not sufficient to prevent glaucoma [5]. Protection against optic nerve damage is also an important factor [6].

Apoptosis has been proven to occur to retinal ganglion cells [7-9]. This mechanism of programmed cell death or cell suicide is initiated when a cell is not necessary or is damaged. It is a physiologic mechanism that allows the organisms to eliminate these cells [10]. Abnormal increases of apoptosis can lead to different disorders.

An increased level of oxidative stress has been reported in glaucomatous optic neuropathy [11,12]. Oxidative stress is a result of imbalance between prooxidants and antioxidants. So in these conditions there are high levels of free radicals that act by damaging cells and can induce the retinal ganglion cell death by apoptosis [13,14]. Glutamate toxicity has also been involved in retinal ganglion cells (RGC) death, so even though IOP is efficient controlled, RGC death will continue if toxic effects of glutamate are not prevented [15,16].

Serotonin is another molecule that is involved in the pathogenesis of glaucoma [17]. It is an indolamine, which is a precursor of melatonin. The potential antioxidant capacity of melatonin is well known [18,19], and there is evidence to suggest that melatonin could decrease the intraocular pressure. Therefore, melatonin could be used in glaucomatous therapy [20]. It is also known that melatonin also has an inhibitor effect on nitric oxide levels, so this molecule could protect cells from nitrosative stress [21].

The study of serotonin, its metabolites, and their role in glaucomatous optic neuropathy could be very important for developing new strategies against this disease. For that reason, we have studied the serotonin (5-HT) and 5-hydroxiindolacetic acid (5-HIAA, a product of serotonin degradation) levels in aqueous humor (AH) of patients with primary open-angle glaucoma (POAG) and patients with cataracts (used as the comparative control group).

## Methods

A case-control study was performed in 60 eyes of 60 patients who were selected from the Department of Ophthalmology of Dr. Peset University Hospital (Valencia, Spain) and Punta de Europa Hospital (Algeciras, Cadiz, Spain) according to the inclusion/exclusion criteria (Table 1) and were classified into two groups, 1) the glaucoma group, which consisted of patients with POAG (GG, n=30) and 2) the cataract group, which consisted of patients with cataracts (CG, n=30).

**Table 1:** Exclusion criteria.

**Glaucoma**	**Cataracts**
Glaucoma different of POAG	Ocular pathology different of cataracts
Age under 41 or over 90 years	Family history of glaucoma
	Age under 41 or over 90 years

POAG=primary open-angle glaucoma.

The Clinical Research Ethics Committee and the Research Committee of the Dr. Peset University Hospital approved this study, which followed the Helsinki guidelines for human research. In all glaucoma patients, the surgical technique used was Watson’s trabeculectomy. The demographic characteristics of participants of both glaucoma and cataract groups are shown in Table 2.

**Table 2:** Features of the studied groups.

**Groups**	**N**	**Age (mean±SD)**	**Gender**		
			**Male**	**Female**	**SR**
GG	30	71.267±7.182	13 (43%)	17 (57%)	0.765
CG	30	73.533±8.989	12 (40%)	18 (60%)	0.667

GG=glaucoma group; CG=cataract group; SD=standard deviation; SR=sex ratio (number of male/number of female).

A sample of AH was obtained from each patient through an anterior chamber paracentesis at the onset of the surgery using a 27 gauge needle under an operating microscope with special care to avoid contamination and was immediately frozen at −85 °C until processing at IBMC-Instituto de Biologia Molecular e Celular (Porto, Portugal).

### Tissue preparation

Human AH samples were diluted 1:3 v/v in 0.2N perchloric acid, filtered through a 0.2 μm Nylon microfilter (Costar, Cambridge, MA) by centrifugation (10,000 rpm for 5 min at 4 °C), and immediately analyzed by high performance liquid chromatography (HPLC).

### High performance liquid chromatography-electrochemical detection equipment and conditions

The high performance liquid chromatography with electrochemical detection (ECD) was applied according to a modified method of Ali [22]. Analyses were performed using a Gilson Medical Electronics HPLC system (Middleton, WI) with a LC-234 auto-injector equipped with a LC307 delivery pump and with a LC142 electrochemical detector under reversed phase conditions with a Supelcosil LC 7.5 cm×4.6 cm, 3 mm column (Supelco; Sigma-Aldrich, Bellefonte, PA). The software used was a 712 HPLC system controller data version 1.30 management (Gilson Medical Electronics). Compounds were eluted isocratically over an 18 min runtime at a flow rate of 1 ml/min. The mobile phase consisted of 70 mM potassium dihydrogen phosphate buffer (pH adjusted to 3.0 with phosphoric acid), 1 mM 1-hepatosulfonic acid, 107.5 mM sodium EDTA, and 10% methanol. Sample injection was 20 ml, and the electrochemical detector was recorded with a glassy carbon working electrode set at +0.75 V.

### Qualitative and quantitative analysis

Identification was performed by comparison with standard retention times determined by injections of standard mixture run at given intervals between sample analyses. Capacity factor was not considered a chromatographic parameter since it was not possible to determine the dead volume under the given HPLC conditions. Quantification was made using the calibration curve standards with 5-HT (r=0.0004) and 5-HIAA (r=0.0003). Samples were injected in duplicate, and the amount of each compound was expressed in ng/ml of AH.

### Statistical analysis

Data were analyzed using SPSS program version 14.0 (SPSS Inc., Chicago, IL). Kolmogorov–Smirnov test was used for checking the normality. Then, Student’s *t*-test for independent samples was used when comparing results. Pearson’s correlation was used to check the correlation between variables. The statistic level of significance was considered at p<0.05.

## Results

**Figure 1:**
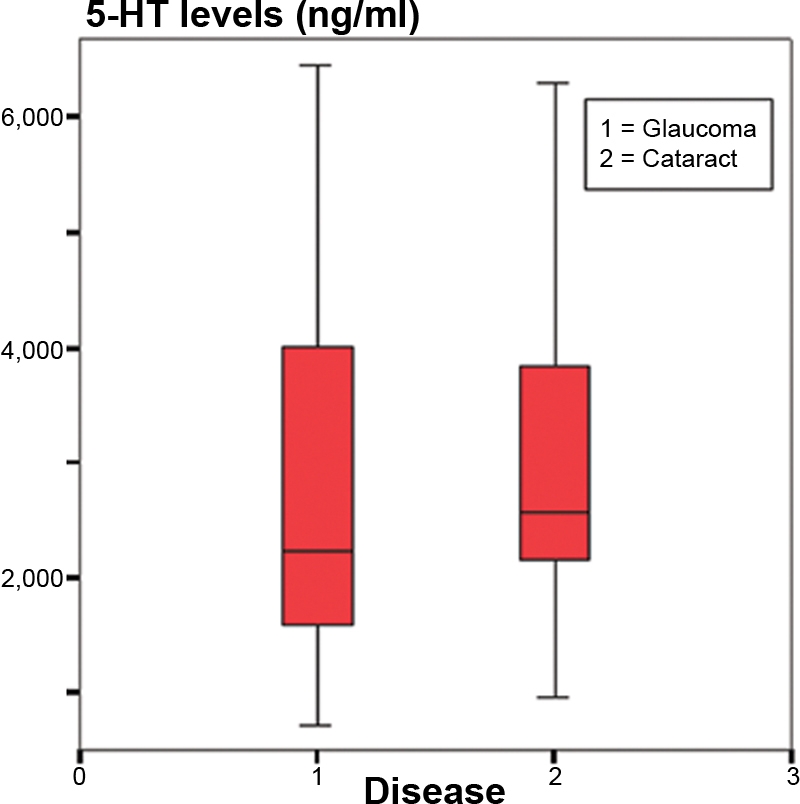
Serotonin levels. The chart shows lower levels of serotonin in aqueous humor of glaucoma patients than in aqueous humor of cataracts patients (2.838 ng/ml in GG and 3.076 ng/ml in CG; p=0.820).

**Figure 2:**
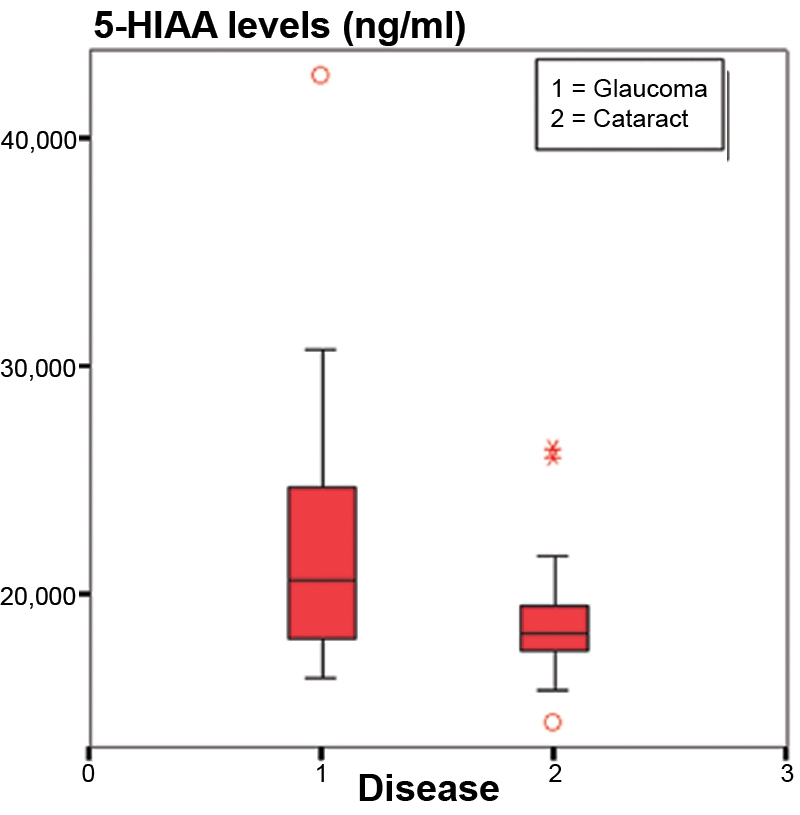
5-HIAA levels. The chart shows statistically significant higher levels of hydroxiindolacetic acid in aqueous humor of glaucoma patients than in aqueous humor of cataracts patients (22.317 ng/ml in GG and 18.816 ng/ml in CG; p=0.001).

**Figure 3:**
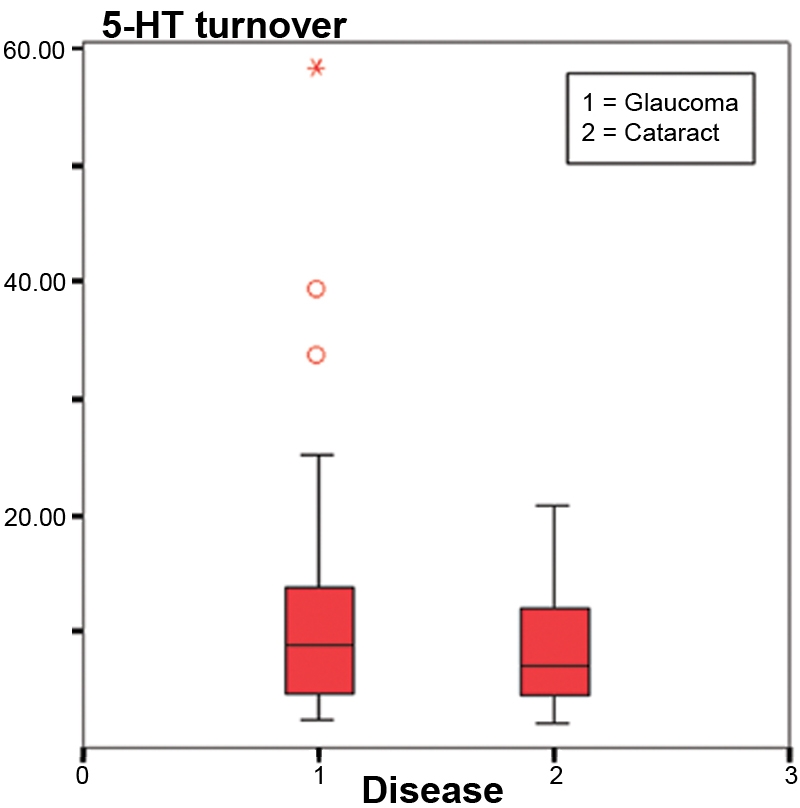
Serotonin turnover. The chart shows the 5-HIAA/5-HT ratio in the aqueous humor of both glaucoma and cataract groups. 5-HT turnover was higher in glaucoma patients than in cataracts patients (14.050 in GG and 12.684 in CG; p=0.598).

**Figure 4:**
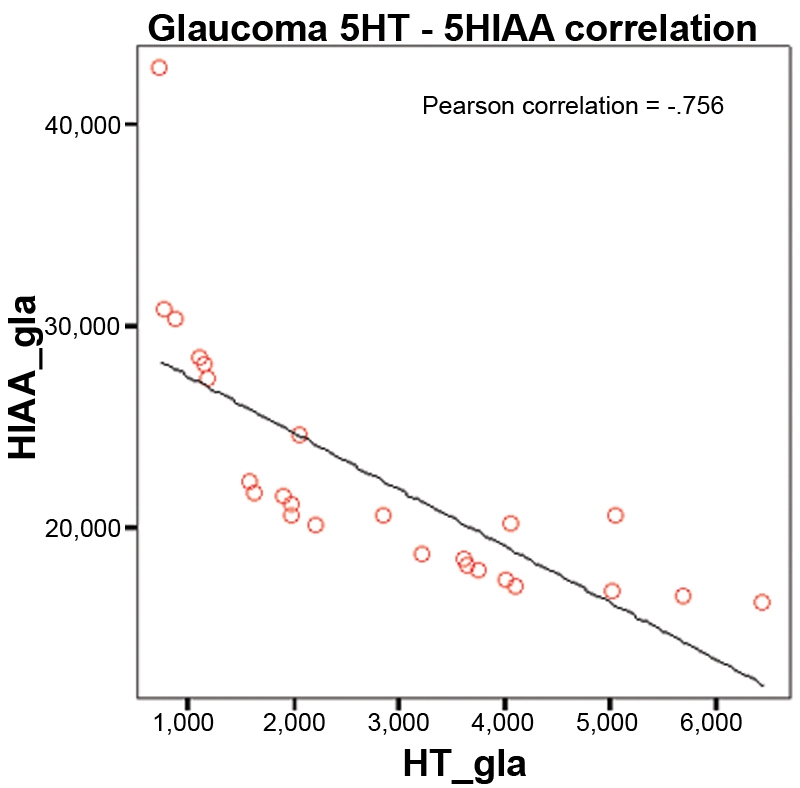
Pearson’s correlation between 5-HT and 5-HIAA in the glaucoma group. The chart shows a statistically significant negative correlation between serotonin and hydroxiindolacetic acid in glaucoma group (p=0.000).

**Figure 5:**
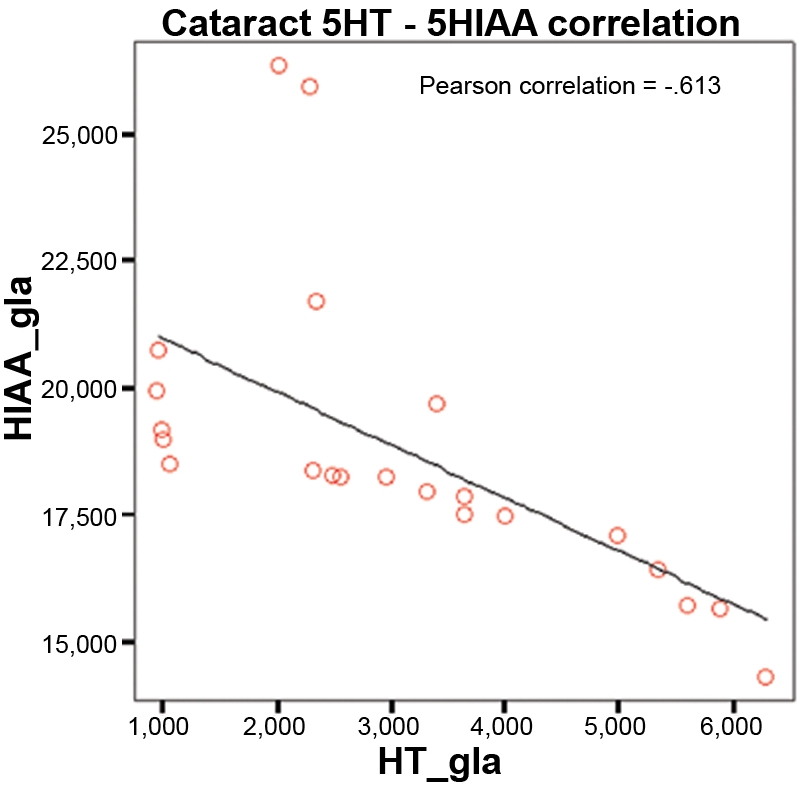
Pearson’s correlation between 5-HT and 5-HIAA in cataract group. The chart shows a statistically significant negative correlation between serotonin and hydroxiindolacetic acid in cataracts group (p=0.002).

No significant age differences were found between both studied groups (71.3±7.2 years in GG, 73.5±9.0 years in CG; p=0.2581) nor any significant gender differences (sex ratio 0.765 in GG and 0.667 in CG; Table 2). We observed higher levels of serotonin in the cataract group than in the glaucoma group (Figure 1), but the difference was not significant (2.838 ng/ml in GG and 3.076 ng/ml in CG; p=0.820). Figure 2 shows that 5-HIAA levels were statistically higher in GG than in CG (22.317 ng/ml in GG and 18.816 ng/ml in CG; p=0.001). The 5-HT turnover (5-HIAA/5-HT) was higher in GG, but the difference was not significant (14.050 ng/ml in GG and 12.684 ng/ml in CG; p=0.598; Figure 3). The correlation between 5-HT and 5-HIAA was assessed by means of Pearson’s correlation (Figure 4 and Figure 5), and the levels of 5-HT and 5-HIAA were associated (glaucoma 5-HT/5-HIAA: Pearson=−0.756; p=0.021; cataracts 5-HT/5-HIAA: Pearson=−0.613; p=0.028).

## Discussion

Glaucoma is an optic neuropathy. Therefore, the study of neurotransmitters and their role in this neurodegenerative disorder is very important for preventing glaucomatous blindness [23]. In this study, we have studied the levels of serotonin in aqueous humor of patients with POAG.

Serotonin is a neurotransmitter that is synthesized in neurons and stored into vesicles. It is present in the mammalian eye, and its levels are higher in the iris-ciliary body complex (ICBC) [24]. After a nervous impulse, 5-HT is released in the synaptic cleft where it interacts with post-synaptic receptors. There is evidence that serotonin plays a role in the regulation of AH dynamics [25]. Seven types of serotonin receptors have been identified (5-HT_1_ to 5-HT_7_). The stimulation of 5-HT_7_ receptor causes an increase in IOP, and the stimulation of 5-HT_1A_ receptor causes a decrease in IOP [26,27]. On the other hand, 5-HT is a precursor of melatonin, a hormone where its concentration varies cyclically and plays a role in a variety of cellular processes such as oxidative stress [28,29].

Oxidative stress is an imbalance between prooxidant and antioxidant molecules and causes toxic effects that damage proteins, lipids, and DNA [30]. It has been related to some ocular diseases like cataracts, age macular degeneration, and glaucoma [31-34].

Serotonin is a precursor of melatonin. The potential antioxidant capacity of melatonin is well known, and there is also evidence that melatonin could decrease IOP [35]. This evidence implies that melatonin may be useful in glaucomatous optic neuropathy [36]. For that reason, we think that 5-HT in POAG patients does not lead to the synthesis of melatonin, but it is degraded by means of monoamine oxidase enzyme (MAO) and the 5-HIAA levels would increase.

The present study results agree with this hypothesis because we have observed higher levels of 5-HIAA in association with lower 5-HT levels in patients with POAG. In addition, some authors have suggested that melatonin has inhibitor effects on nitric oxide levels [37,38]. Therefore, melatonin levels in these patients should be low. We have evaluated in another study the nitric oxide levels in patients with POAG, and the results have shown an increase of nitric oxide with respect to the comparative group [37]. All these findings showed the relation of 5-HT to the ethiopathogenic mechanisms of primary open-angle glaucoma and could be used in the design of new therapies for the early diagnosis and prevention of glaucomatous blindness.
